# Functions, Characteristics, and Experiences of Non-Suicidal Self-Injury: A Cross-Sectional Study of Youth and Adolescents in Singapore

**DOI:** 10.1192/bjo.2024.129

**Published:** 2024-08-01

**Authors:** Xiaowen Lin, Michelle Hui Ling Neo, Jamie Jiehan Ong, Ying Jie Fong, Tji Tjian Chee

**Affiliations:** 1 National University Singapore, Singapore, Singapore; 2 National University Hospital, Singapore, Singapore

## Abstract

**Aims:**

Non-Suicidal Self-Injury (NSSI) occurs when direct, deliberate harm is caused to one's physical body without intention of suicide. Approximately 22.1% of youth worldwide would engage in NSSI in their lifetimes. Due to the increased risk of harm and future suicide attempts, NSSI is a behaviour that warrants attention and has been identified as a condition in need of further study. While some studies have examined the prevalence and experiences of NSSI in Singapore, there is a lack of detailed studies on the presentation and overall phenomenology of NSSI in the local context. This study aims to assess the characteristics of NSSI using the Non-Suicidal Self-Injury – Assessment Tool (NSSI-AT) in a cross-sectional design. We investigated the functions, characteristics, and personal experiences of local youths who engage in NSSI for the development and improvement of patient-centred care.

**Methods:**

121 youths between 12 and 25 years old were recruited from the National University Health System. The study included patients seeking treatment for mood disorders and have self-reported NSSI behaviours such as cutting, hitting, and scratching prior to or at the time of visit. Outcomes for the NSSI–AT, including the actions, functions, frequency, age of onset, initial motivations, severity, practice patterns, disclosure, and treatment experiences of self-harm, were reported using descriptive analysis. Personal reflections were analysed using thematic analysis.

**Results:**

Participants were mostly female (n = 86, 71.1%) with a mean age of 16.2 years (*SD* = 2.33). Many participants engaged in NSSI actions such as cutting, scratching, and banging on objects, to manage high-pressure agitating and low-pressure depressive emotional states. Most participants started engaging in NSSI in early adolescence (mean = 13.0 years old, *SD* = 2.37, range = 7–23) and have hurt themselves more severely than intended (n = 79, 65.3%). When reflecting on overall NSSI experiences, participants had similar levels of ambivalence toward NSSI and growth due to NSSI. Participants also gave encouragement to others going through similar experiences and reported the negative aspects of self-harm.

**Conclusion:**

Findings support emotional regulation as a function of NSSI in the local population, where self-harm was not generally used for social communication purposes. Findings also suggest that youths may be more vulnerable to NSSI during early adolescence, corresponding to a time of substantial life changes. This study also demonstrated the individuality of NSSI experiences among the local youth, highlighting the importance of having a person-centred approach in NSSI treatment. Taken together, this highlights the need to develop interventions that can effectively serve this age group and their specific challenges.

